# Investigation into the Adsorption of Methylene Blue and Methyl Orange by UiO-66-NO_2_ Nanoparticles

**DOI:** 10.1155/2021/5512174

**Published:** 2021-06-12

**Authors:** Hien Thi Dinh, Nam Trung Tran, Dai Xuan Trinh

**Affiliations:** Faculty of Chemistry, VNU University of Science, Vietnam National University, 19 Le Thanh Tong, Hoan Kiem, Hanoi, Vietnam

## Abstract

In this work, the adsorptive removal of methylene blue and methyl orange by UiO-66-NO_2_ nanoparticles was studied. The influence of pH on the adsorption capacity was assessed. The kinetics of the adsorption process were investigated and compared with pseudo-first-order, pseudo-second-order, Elovich, and intraparticle models. The kinetics of the adsorption fits moderately with the pseudo-first-order, but perfectly fits with pseudo-second-order models, and has a very good fit with the Elovich and intraparticle models. The adsorption isotherms were measured and compared with the Langmuir and Freundlich models. The adsorption capacity of methyl orange (MO) on UiO-66-NO_2_ nanoparticles (142.9 mg/g) was over three times higher than that of methylene blue (MB) on the nanoparticles (41.7 mg/g). The discrepancy between these capacities was attributed to the presence of the -NO_2_ functional group, which caused a strong negative mesomeric effect in the metal-organic framework structure.

## 1. Introduction

Water is unarguably an indispensable part to maintain human lives on earth. Therefore, it should be protected and used rationally. However, the water source has been potentially damaged by different activities of humans. Among these, it is agreed that the textile industry consumes a huge amount of fresh water for the production [1]. Importantly, during the production, different types of dyes are used [2]. These compounds can pollute into the water body if wastewater from the textile industry is not treated adequately [3]. The resulting pollution poses different adverse effects to the creatures living in the water body. The presence of dyes in water hinders the photosynthetic processes, thus lowering the concentration of dissolved oxygen. Low dissolved oxygen concentration can lead to different types of diseases and, in worse cases, to the death of the living creatures therein [4–6]. Moreover, many dye compounds are considered as potentially mutagenic, carcinogenic, and highly toxic [7–9]. The intake of water heavily contaminated with these dyes causes diseases in humans, for instance, allergy, central nervous system disorder, and cancer. These diseases are related to different metabolisms of dye molecules in the human body [10]. Dye molecules can substitute enzymatic cofactors in humans, thus inactivating the function of the enzymes. They can also form conjugates with human serum albumin. Additionally, they can have strong interaction with DNA, which leads to the damages and distortion of DNA [11]. These issues are worsened due to the persistence of the dyes in the environment [12]. Several dyes were found to have a half-life of up to 13 years [13]. Several others might be degraded faster, but the decomposition process gives rise to the formation of mutagenic and carcinogenic intermediate molecules [14].

Due to the persistence of the dye molecules, they are not degradable during the biological treatment of a conventional wastewater treatment plant. Therefore, special treatment methods are required for these compounds. Suitable chemical processes include ozonation, Fenton oxidation, photochemical treatment, oxidation with hyperchlorite, and electrochemical destruction [15–17]. On the contrary, physical methods to treat dyes include membrane filtration and adsorption [15–21]. Adsorption is considered as a fast method to treat dye pollution which effectively works with different types of dyes. Among different adsorbents used for this method, metal-organic framework (MOF) materials have attracted increasing attention due to their particularly high specific surface areas [22–24].

Metal-organic frameworks (MOFs) are porous material class composed of inorganics salts and multidentate organic linkers. Metal-organic frameworks have been widely investigated for the recent two decades because of their advantages such as superior high surface area, tunable texture, and structure diversity, which make MOF suitable for gas and liquid adsorption and separation, catalysis, drug delivery, and wastewater treatment processes. In terms of dye adsorption, MOFs have emerged as promising materials. Muhamet et al. reported that Cu-BTC was a superior adsorbent for the removal of methylene blue with the adsorption capacity about 200 mg/g [25]. In another research, Ying et al. synthesized a 3D anionic MOF to adsorb rhodamine B and methylene blue, and they found that the adsorption capacity of the material reached 100 mg/g [26]. Zha et al. reported that a superhydrophobic MOF exhibited enhanced dye adsorption ability, and the adsorption capacity of the hydrophobic MOF reached 478 mg/g compared to 243 mg/g of the pristine hydrophilic MOF [27]. These results opened an emerging class of materials for dye adsorption.

Zirconium-based MOF family has been known as the most stable MOFs because of their largest coordination between the metal node and the organic linker, which prevents the metal node from the attack of chemicals. As a result, the MOFs maintain their structure in the presence of solvents such as water, ethanol, DMF, and chemicals. Among members of the family, UiO-66 has been investigated the most in adsorption applications, and most of them exhibit the outstanding performance of UiO-66. However, Chen et al. investigated the adsorption ability of UiO-66-NH_2_ to dyes and reported that the electrostatic interaction between the functional group of the adsorbent and the adsorbate drastically governs the adsorption capacity of the materials [28]. In other words, the functional groups play a crucial role in the selective adsorption ability of MOFs. Apart from containing the functional group, UiO-66-NO_2_ has been known as the MOF which is stable in the broadest range of pH [29], which endows UiO-66-NO_2_ an ability in a broad pH range application.

**Figure 1:**
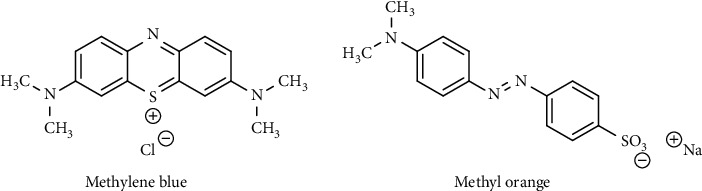
Chemical structures of methylene blue (MB) and methyl orange (MO).

In this research, for the first time, UiO-66-NO_2_ was applied to remove the dyes. The characterization of UiO-66-NO_2_ was performed using the FT-IR, XRD, TGA, and nitrogen adsorption/desorption techniques. The adsorption performance of the MOF was investigated on the removal of methylene blue (MB) as a cationic dye and methyl orange (MO) as an anionic dye whose structures are given in Figure 1. The Langmuir and Freundlich isotherms were examined, and the adsorption capacity and kinetics of the adsorption process were also performed. The research may offer a promising material for industrial applications.

## 2. Experimental

### 2.1. Chemicals

Zirconium tetrachloride (ZrCl_4_, purity > 99.9%) was purchased from Sigma-Aldrich. 2-Nitroterephthalic acid (purity > 98.0%) was obtained from Acros Organics. Dimethylformamide (DMF) was received from Wako Chemical Industries Ltd. Methylene blue (MB, purity > 98.0%) and methyl orange (MO, purity > 98.0%) were bought from TCI Chemicals Ltd. These chemicals were employed without further purification.

### 2.2. Characterization

Transmission FT-IR spectra were acquired in KBr pellets using a Jasco FTIR-6100 spectrometer. The measurement was carried out in the range of 450–1650 cm^−1^. X-ray diffraction (XRD) was analyzed on a Rigaku SmartLab diffractometer using Cu K*α* radiation with *λ* = 1.54 Å at 40 kV and 30 mA in the interval 2*θ* = 5–40°. Thermal gravimetric analysis (TGA) was performed on Mettler Toledo DSC 820 under air atmosphere in the range of 30–700°C at a heating rate of 5°C·min^−1^. The nitrogen adsorption-desorption analysis was conducted on NOVAtouch LX4 at 77 K. The sample was dried at 100°C in a vacuum oven for 3 h before the measurement.

### 2.3. Preparation of UiO-66-NO_2_ Nanoparticles

UiO-66-NO_2_ nanoparticles were prepared according to previous studies [30, 31]. In a typical procedure, 0.28 g of 2-nitroterephthalic acid and 0.22 g of zirconium tetrachloride were dissolved in 60 ml of DMF under nitrogen atmosphere. The solution was transferred into a Teflon-lined autoclave before being heated at 100°C for 24 h. UiO-66-NO_2_ nanoparticles were obtained as precipitates which were washed several times with methanol before being dried at 100°C in a vacuum oven for 24 h.

### 2.4. Adsorption Procedure

10.0 ml of a dye solution of a chosen concentration between 20 and 400 mg·L^−1^ was subjected into an Erlenmeyer flask. To the solution, 0.01 g of UiO-66-NO_2_ adsorbent was added before being well shaken. After a certain time, an aliquot was taken from the solution. The concentration of the dye in the aliquot was measured by UV-Vis spectroscopy at 665 and 465 nm for MB and MO, respectively. The concentration of the dye adsorbed onto UiO-66-NO_2_ was calculated as the following:

(1) $$\begin{array}{c} q\;=\;\frac{C_{0}-C_{t}}{M}\cdot V, \end{array}$$

where *C*_0_ is the initial dye concentration (mg·l^−1^), *C*_*t*_ is the dye concentration at time *t* (mg·L^−1^), *M* is the amount of UiO-66-NO_2_ (g), and *V* is the volume of the solution (L).

The removal percentage of the dye was calculated as the following:

(2) $$\begin{array}{c} R\;=\;\frac{C_{0}-C_{t}}{C_{0}}\cdot 100\%, \end{array}$$

where *C*_0_ is the initial dye concentration (mg·L^−1^) and *C*_*t*_ is the dye concentration at time *t* (mg·L^−1^).

## 3. Results and Discussion

### 3.1. Characterization of UiO-66-NO_2_ Nanoparticles

**Figure 2:**
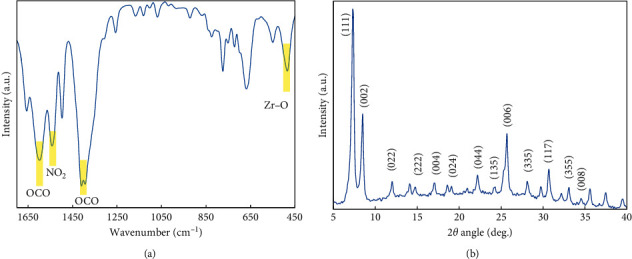
FTIR spectra (a) and XRD diffractogram (b) of prepared UiO-66-NO_2_ nanoparticles.

The prepared UiO-66-NO_2_ nanoparticles were analyzed by FTIR spectroscopy (Figure 2(a)). The spectrum displays typical bands responsible for the functional groups C=O (1597 and 1400 cm^−1^), NO_2_ (1544 cm^−1^), and Zr-O (484 cm^−1^). Compared to the bands of UiO-66 materials [32], those of UiO-66-NO_2_ material were slightly shifted to the lower wavelength. The reason for this shift is probably due to the negative mesomeric effect resulted by the -NO_2_ groups in the structure of UiO-66-NO_2_ material, which weakens the corresponding C=O and C=C bonds.

**Figure 3:**
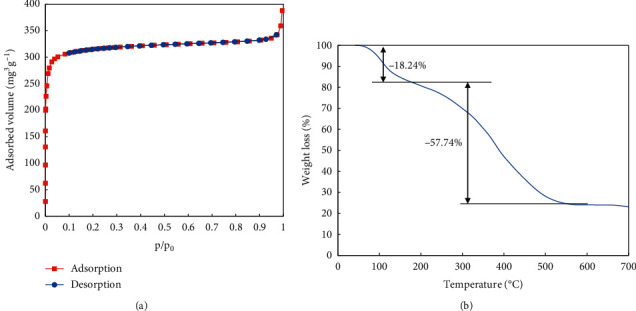
TGA diagram and nitrogen adsorption isotherm of the prepared UiO-66-NO_2_ material.

On the contrary, the XRD diffractogram of UiO-66-NO_2_ material shows the typical peak of the face-centred cubic structure of the UiO-66 (Zr)-based metal-organic framework family [31, 33–35] such as peaks at 7.3, 8.4, and 11.9° corresponding to the (111), (002), and (022) planes, respectively (Figure 2(b)). In addition, the TEM image of UiO-66-NO_2_ material shows particles with size in the range 45–65 nm (Figure S1, Supplementary Materials). The small size of the material allows maximal contact with the adsorbents, which is beneficial for the adsorption process. The nitrogen sorption isotherm of UiO-66-NO_2_ material is displayed in Figure 3(a). It represents an isotherm categorised as type I, which is a characteristic for microporous materials. The BET specific surface area is 970.2 m^2^·g^−1^, which is higher than the value of the UiO-66-NO_2_ material in the study carried out by Rada et al. (771.0 m^2^·g^−1^) [36]. However, it is lower than the value of UiO-66-NH_2_ in the same study (1025 m^2^·g^−1^). In the study of Song et al. [37], the BET surface of UiO-66 was found up to 1684 m^2^·g^−1^. Noticeably, this value decreases with an increasing amount of water and solvent contents, which remain in the pores of the material. The positions filled with water and solvent molecules are considered as defects of the material structure [38, 39].

The TGA diagram of UiO-66-NO_2_ is displayed in Figure 3(b). As can be seen, from 40 to 174°C, a loss of 18.2 wt% was observed, which is responsible for the removal of water and ethanol from the material. The subsequent loss is assigned to the deviation of DMF molecules adsorbed on the surface of the material. After this loss, another weight loss of 57.7% was observed from 190 to 512°C, which is corresponding to the rupture of the benzene and carbonyl groups. After 512°C, the material shows a residue of 24.0% which is assigned to the remaining ZrO_2_.

### 3.2. Adsorption of Methylene Blue and Methyl Orange onto UiO-66-NO_2_ Nanoparticles

#### 3.2.1. Adsorption Kinetics

In principle, the adsorption of dyes can be divided into at least four major stages. The first stage involves the mass transfer of dye molecules towards the adsorbents (bulk movement). Unless stirring is set at particularly low speeds, this stage is a fast process which takes place right after the addition of the adsorbents into the aqueous solution. When the dye molecules are about the adsorbents, the second stage is initiated, which involves a slow diffusion of these molecules towards the surface of the adsorbents (film diffusion). It is followed by the third stage, which is a slow diffusion of the dye molecules into the pores of the adsorbents (intraparticle diffusion). During the final stage, the dye molecules are rapidly anchored to the active sites of the adsorbents. Generally, the film and the intraparticle diffusions qualitatively determine the rate of the adsorption of the dyes onto the adsorbents. Among them, one factor might be more predominating than the other in certain cases. At low dye concentration, the film diffusion is more predominating, while the intraparticle diffusion has a greater impact on the rate of the adsorption in many other cases.

**Figure 4:**
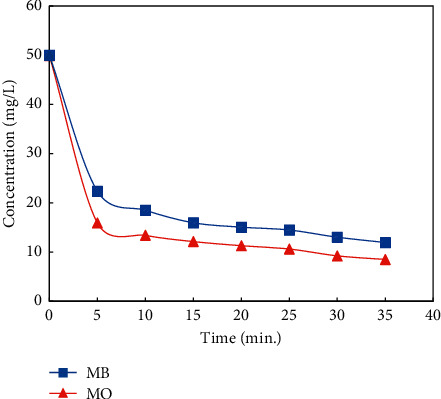
Adsorption of methylene blue (MB) and methyl orange (MO) by the prepared UiO-66-NO_2_ material.

The adsorption of MB and MO onto UiO-66-NO_2_ is demonstrated in Figure 4. As can be seen, the fast bulk movement was observed in the time from 0 to 5 min, when the concentration of MB and MO was drastically reduced from 50.0 to 22.3 and 15.9 mg/L, respectively. From 5 to 35 min, the concentration of MB and MO was reduced gradually, being controlled by the film and intraparticle diffusions.

To quantitatively describe the kinetics of the adsorption of MB and MO, four popular approaches were applied, namely, (a) pseudo-first-order (PFO), (b) pseudo-second-order (PSO), (c) Elovich, and (d) intraparticle diffusion models. Each of these models makes individual assumption about the nature of the adsorption process to enable a quantitative calculation of the kinetics. The corresponding mathematical equation was transformed into linear forms, for which regressions with the experimental data were calculated. Finally, the correlation coefficient *R*^2^ was used to evaluate the correlation of the experiment data with the kinetics model.

In the PFO model, the kinetics of the adsorption is assumed to be proportional to the number of active sites of the adsorbent and reciprocal to the concentration of the dyes. The corresponding kinetic equation is given as the following:

(3) $$\begin{array}{c} \log\left(q_{e}-\;q_{t}\right)=\log\;q_{e}-\frac{k_{1}}{2.303}t, \end{array}$$

where *q*_*t*_ represents the amount of dyes adsorbed onto the adsorbent (mg·g^−1^), *q*_*e*_ is the equilibrium adsorption capacity (mg·g^−1^), and *k*_1_ is the rate constant (min^−1^).

Applying the experimental data into equation (3), the relationship between these parameters for MB is given in Figure S2. As can be seen, at the concentration of 50 and 75 mg/L, there were linear relations following pseudo-first-order reaction kinetics. However, at the concentration of 100 mg/L, the correlation coefficient was only 0.83 which suggests that the pseudo-first-order reaction kinetics was irrelevant to adsorption of MB onto UiO-66-NO_2_. It is notable to mention that, in this case, from 5 to 20 min, a perfect linearization was observed (*R*^2^ = 0.99), which was assumed that the process from 5 to 20 min follows the pseudo-first-order reaction kinetics. Nevertheless, the linearization was deviated from 25 min driving the overall correlation coefficient as low as 0.83. Therefore, the pseudo-first-order reaction kinetics may be applicable to the adsorption at a low MB uptake, and at high MB amount adsorption, the pseudo-first-order kinetics was inappropriate.

Applying the experiment data into equation (3) for MO, the relationship between the parameters of the PFO model is derived, being given in Figure S3. There was a good fit of the MO adsorption to the pseudo-first-order reaction kinetics in which the correlations for 50, 75, and 100 mg/L were 0.95, 0.95, and 0.98. From the linear regression, we conclude that the PFO model gives a quite representation of the experimental data for MO adsorption.

In comparison to PFO, the PSO model assumes that the rate of the adsorption process is proportional to the available active sites of the adsorbent which follows the equation

(4) $$\begin{array}{c} \frac{\text{d}q_{t}}{{\text{d}}_{t}}=k_{2}\cdot {\left(q_{e}-q_{t}\right)}^{2}. \end{array}$$

By taking the corresponding integral, the linear form of the equation is derived as the following:

(5) $$\begin{array}{c} \frac{t}{q}\;=\;\frac{1}{k_{2}q_{e}^{2}}+\;\frac{t}{q_{e}}. \end{array}$$

Applying the experiment data into the linear form of equation (5), we obtained a very good correlation (*R*^2^ = 0.99) for the entire adsorption of MB and MO (Figures S4 and S5). These data suggested that the chemisorption was more preferential than physisorption for the adsorption of both dyes onto the UiO-66-NO_2_ nanoparticles [40]. *k*_2_ was the rate of adsorption of the dyes which revealed that the adsorption of MB (*k*_2_ = 0.064 g/mg·min) was faster than that of MO (0.034 g/mg·min) on the nanoparticles.

The Elovich model assumes that the rate of the adsorption decreases in an exponential manner with an increase in the number of dye molecules, following a chemisorption mechanism [41]. The kinetics for this model is described as the following:

(6) $$\begin{array}{c} \frac{\text{d}q_{t}}{\text{d}t}=\alpha \cdot e^{-\beta q_{t}}. \end{array}$$

After taking the integral and approximation conditions, *q*_*t*_ ≈  0, *dq*_*t*_/*dt* ≈  *α*, and *t* ≫ 1/*α* · *β*, and the corresponding linear form of equation (7) is derived:

(7) $$\begin{array}{c} q_{t}\;=\;\frac{1}{\beta }\cdot \;\ln\left(\alpha \beta \right)\;+\;\frac{1}{\beta }\cdot \;\ln\;t. \end{array}$$

Applying the experiment data of MB into the linear form of equation (7), the relationship between the reaction parameters is demonstrated in Figures S6 and S7. As can be seen, the Elovich model gives a very good fit with the experimental data of MO in which *R*^2^ was varied in the range of 0.92 to 0.99. On the contrary, the Elovich model gives a moderate fit with the experimental data of MB with *R*^2^ ranging from 0.85 to 0.97. The good fit of the experimental data of MO with Elovich indicates the existence of the chemisorption interaction between this dye and the adsorbent.

In the intraparticle diffusion model, the effect of the boundary layer is considered. The application of this model might give hints whether the intraparticle diffusion is the predominant rate-determining factor. The equation of the intraparticle diffusion model is given as the following [42]:

(8) $$\begin{array}{c} q_{t}\;=\;k\cdot t^{1/2}\;+\;C. \end{array}$$

Therefore, we applied the experimental data to examine the linear relationship between the variable *q*_t_ and *t*^1/2^ in equation (8). The results are demonstrated in Figures S8 and S9. As can be seen, the intraparticle model gives a very good fit for MB (*R*^2^ from 0.91 to 0.98) and excellent fit for MO (*R*^2^ within 0.92–0.97). It indicates that, in both cases, the intraparticle diffusion is the rate-determining stage of the adsorption.

#### 3.2.2. Adsorption Isotherm

**Figure 5:**
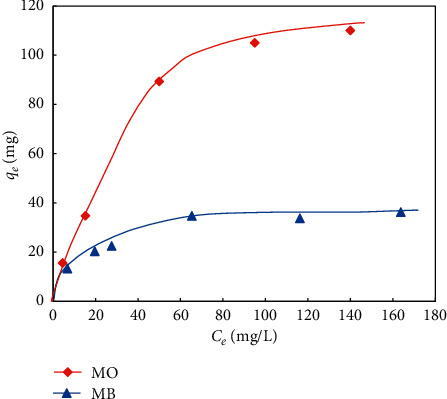
Langmuir adsorption isotherms of MO and MB adsorption onto UiO-66-NO_2_.

The adsorption isotherms exhibited the MO and MB adsorption onto UiO-66-NO_2_ which are represented by the Langmuir isotherms as the following equation [43] and displayed in Figure 5.

(9) $$\begin{array}{c} q_{e}=\frac{q_{\max}K_{L}C_{e}}{1+\;K_{L}C_{e}}, \end{array}$$

where *q*_*e*_ represents the equilibrium dye concentration in the absorbents (mg·g^−1^), *C*_*e*_ is the equilibrium dye concentration in the solution (mg·l^−1^), *q*_max_ is the monolayer adsorption capacity of the adsorbents (mg·g^−1^), and *K*_*L*_ is the Langmuir adsorption constant (l·mg^−1^).

As seen in Figure 5, at the low adsorbate concentration, when the active sites for the adsorption were abundant on the adsorbent surface, the amount of MO adsorbed on the adsorbent sharply increased till the active adsorption sites on the adsorbent decrease before reaching the saturation state. The curve for MB adsorption was in the same scenario. However, the slope for adsorption increment at the low concentration step was significantly lower than that of MO adsorption. Also, the maximum adsorption of UiO-66-NO_2_ for MB was far less than for MO dye. From the adsorption isotherms, the maximum adsorption capacity of MO on UiO-66-NO_2_ was about 115 mg/g, while the maximum value for MB was about 36 mg/g. These data revealed that the adsorption of MO on the material was more favourable than that of MB.

**Figure 6:**
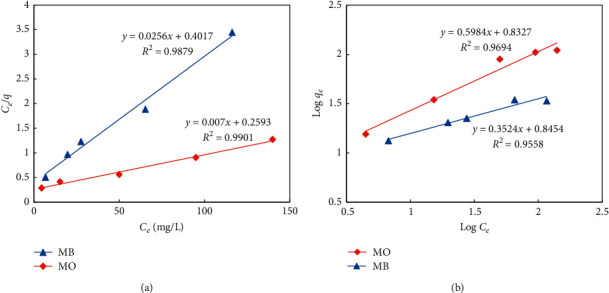
Linear isotherm models for the adsorption of MB and MO onto UiO-66-NO_2_.

To gain a more insightful view about the mechanism of the adsorption, we analyzed their isotherms by applying linear Langmuir and Freundlich models which are depicted in Figure 6.

The Langmuir model assumes that the adsorption of solutes onto adsorbents proceeds via the formation of a single homogenous layer of dye molecules on the surface of the adsorbent. The corresponding equation for the Langmuir isotherm is given as the following [44]:

(10) $$\begin{array}{c} \frac{C_{e}}{q_{e}}=\frac{1}{q_{\max}K_{L}}+\frac{C_{e}}{q_{\max}}. \end{array}$$

Based on this equation, the linear regression between *C*_*e*_/*q*_*e*_ and *C*_*e*_ was analyzed, and the correlation coefficient *R*^2^ and the variable 1/*q*_max_ were estimated. The *R*^2^ values for MB and MO were 0.99 in both cases, indicating that the Langmuir model can be used to precisely describe the kinetics of the adsorption of MB and MO onto UiO-66-NO_2_. The maximum monolayer adsorption (*q*_max_), which represents the adsorption capacity of the adsorbent, was calculated as 41.7 and 142.9 mg·g^−1^ for MB and MO, respectively. Interestingly, the maximum adsorption capacities of dyes on the materials estimated from linear and nonlinear models were inconsistent. The reason for the deviation was explained by the propagated error caused by the least square regression during linearization [43, 45].

The adsorption of dye on the metal-organic framework was reported to depend on the pore sizes of the adsorbents in some previous studies. In the study of Wu et al., the adsorption capacity of MO in ZIF-8 was particularly low despite its high BET specific surface area [46]. It is due to the particularly small pore size of the adsorbent that does not allow the penetration of the MO molecules into the inner pores. For this reason, the adsorption can only take place outside of the pores, for example, in the interspaces between the particles. The comparison of the adsorption capacity of this study with the results of previous studies indicates that the adsorption of MB and MO takes place in both intraparticle and interparticle spaces of UiO-66-NO_2_. Furthermore, the *R*_*L*_ value for MB and MO is estimated as 0.05 and 0.047, respectively, indicating that the process is favourable in both cases. The *R*_*L*_ value for MO is slightly lower than that of MB, suggesting that the adsorption of MO is more favourable than MB. It is different from the results of several previous studies, in which the adsorption capacity of MB is higher than that of MO. The difference is probably related to the presence of the -NO_2_ group with a strong negative mesomeric effect in the structure of UiO-66-NO_2_.

In comparison to Langmuir, the Freundlich model considers the heterogeneity of the surface of the adsorbents such as the repulsion between the adsorbed dye molecules. The Freundlich model is an empirical approach which follows the equation

(11) $$\begin{array}{c} \log\;q_{e}=\log\;K_{F}+\frac{1}{n}\cdot \;\log\;C_{e}, \end{array}$$

where *q*_*e*_ is equal to the equilibrium concentration of dyes in the absorbents (mg·g^−1^), *C*_*e*_ is the equilibrium concentration of dyes in the solution (mg·l^−1^), and *K*_*F*_ (l·g^−1^) and *n* are the Freundlich adsorption isotherm constants. Based on this equation, the linear regression between log *q*_*e*_ and log *C*_*e*_ was analyzed. The *R*^2^ values for MB and MO were estimated as 0.95 and 0.96, respectively. They indicate that the Freundlich model can also be used to precisely describe the adsorption of MB and MO onto UiO-66-NO_2_. The 1/*n* value for MB and MO is 0.31 and 0.60, respectively. This value for MB is lower than MO, indicating that the adsorption of MB is more heterogeneous than MO. This might be attributed to the difference in the adsorption mechanisms of these dyes.

In comparison with adsorption capacities of other MOFs in the zirconium-based family, according to Chen et al., the adsorption capacities of MB on UiO-66 and UiO-66-NH_2_ were 90.88 and 96.45 mg/g, respectively, while for MO, the adsorption capacities on UiO-66 and UiO-66-NH_2_ were 39.42 and 28.97 mg/g. However, the adsorption capacity of MO on UiO-66-NO_2_ was outperformed than that of MB (Table 1). Therefore, the behavior of UiO-66 and UiO-66-NH_2_ with cationic dye and anionic dye was reversed to that of UiO-66-NO_2_. This inversion in the adsorption ability of the MOFs for the dyes might result from the effect of functional groups (-NH_2_ and -NO_2_) on the electrostatic nature of the frameworks, which in turn governed the electrostatic attraction/repulsion of the frameworks with the dyes.

#### 3.2.3. Influencing Factors on the Adsorption of MB and MO onto Adsorbents

**Figure 7:**
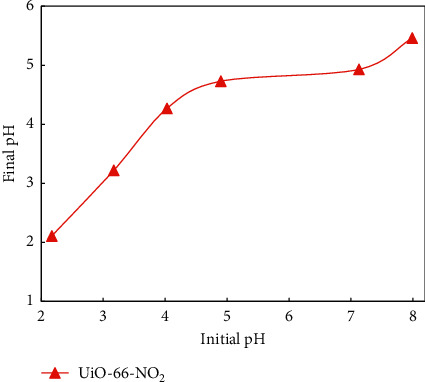
Determination of the point of zero charge (pH_pzc_) of UiO-66-NO_2_.

At first, the influence of pH on the adsorption of MB and MO was studied. pH of the solution can determine the nature of the surface of the adsorbents. The pH point of zero charge (pH_pzc_) was estimated by finding the pH value, in which pH_initial_ is equal to pH_final_ (Figure 7). Below pH_pzc_, the surface of the adsorbents is protonated, thus being positively charged. Positively charged surface facilitates the anchor of anion dyes. In contrast, above the pH_pzc_ value, the surface of the adsorbents is anchored to hydroxide ions, thus being negatively charged. Negatively charged surface facilitates the anchor of cation dyes. In this study, the pH_pzc_ value of the adsorbent was about 4.6 (Figure 7).

**Figure 8:**
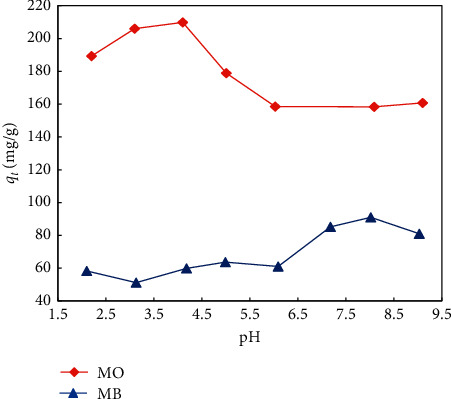
Influence of pH on the adsorption capacity of MB and MO by UiO-66-NO_2_.

Generally, the interactions between UiO-66-NO_2_ and dye molecules can be based on hydrogen bonds, electrostatic attraction, electrostatic repulsion, van der Waals forces, *π*-*π* stacking, and acid-base interaction. The influence of pH on the adsorption capacity of MB and MO onto UiO-66-NO_2_ is demonstrated in Figure 8.

The adsorption capacity of MO onto UiO-66-NO_2_ is given in red color. As can be seen, from pH 2.0 to 4.0, the adsorption capacity of MO decreases from 37.0% to 26.0%. In acidic conditions, the surface of UiO-66-NO_2_ is positively charged where the carbonyl groups of UiO-66-NO_2_ are protonated. The protonation hydrogen atoms can initiate further hydrogen bonds with the MO molecules. The hydrogen bonds and the electrostatic interaction enhance the attraction between UiO-66-NO_2_ and MO molecules. The increase of pH from 2.0 to 4.0 reduces the number of positive charges and the protonation hydrogen atoms, which reduces the adsorption capacity of MO onto UiO-66-NO_2_. The pK_a_ constant for MO is about 3.7 [47]. It means that, at pH above 3.7, the majority of the MO molecules exist in the negatively charged form. From pH 4.0 to 6.0, the surface of UiO-66-NO_2_ is quite neutral (Figure 7). Therefore, in this pH range, the interaction between UiO-66-NO_2_ and MO molecules is majorly based on the nonelectrostatic interaction, such as *π*-*π* stacking and van der Waals forces. From pH 6.0 to 9.0, the adsorption capacity of MO onto UiO-66-NO_2_ almost remains, indicating that the electrostatic repulsion between the negatively charged surface of UiO-66-NO_2_ and MO molecules is negligible. It further indicates that the hydrogen bond interaction was predominant over electrostatic attraction in the pH range of 2.0 to 4.0. Overall, the adsorption between UiO-66-NO_2_ and MO was majorly based on the nonelectrostatic interactions such as *π*-*π* stacking and van der Waals forces. The adsorption can be enhanced owing to the formation of hydrogen bond interactions, which is clearly observed in acidic conditions and partly observed in basic conditions.

The adsorption capacity of MB onto UiO-66-NO_2_ is given in blue color. The pK_a_ constant of MB is 3.8 [48]. At pH lower than this value, the molecules exist majorly in the positively charged form. On the contrary, at pH above this value, the molecules exist majorly in the neutral form. From pH 2.0 to 3.0, the adsorption capacity of MB is decreased. In this range, both UiO-66-NO_2_ and MB molecules exist majorly in the positively charged form. The carbonyl groups of UiO-66-NO_2_ are protonated, which undergo hydrogen bond interaction with the nitrogen atoms of MB molecules. The hydrogen bond interaction is stronger than the electrostatic repulsion. Therefore, the increase of pH from 2.0 to 3.0 reduces the amount of the protonation hydrogen atoms, which reduces the adsorption capacity of MB onto UiO-66-NO_2_. From pH 3.0 to 5.0, the adsorption of MB onto UiO-66-NO_2_ increases. In this pH range, the charge of MB changes from positive to neutral, which eliminates the electrostatic repulsion between UiO-66-NO_2_ and MB molecules that improves the adsorption capacity. From pH 5.0 to 6.0, the adsorption of MB onto UiO-66-NO_2_ was reduced. In this range, the charge of UiO-66-NO_2_ changes from positive to negative, which results in the electrostatic repulsion with the negatively charged MB molecules. Upon increasing pH from 6.0 to 8.0, the adsorption of MB onto UiO-66-NO_2_ increases. During this, the surface charge of UiO-66-NO_2_ changes from positive to negative, indicating the key role of the electrostatic interaction between negatively charged UiO-66-NO_2_ and the MB molecules with partially positive charges. However, the overall impact of the hydrogen bond interaction between UiO-66-NO_2_ and MB molecules is still predominant, but less than in case of MO. Therefore, the impact of the electrostatic repulsion and attraction was seeable in some cases. It also indicates that the nonelectrostatic interaction in case of MO is remarkably higher than in case of MB, which partially explains the higher *q*_max_ value of MO over MB.

**Figure 9:**
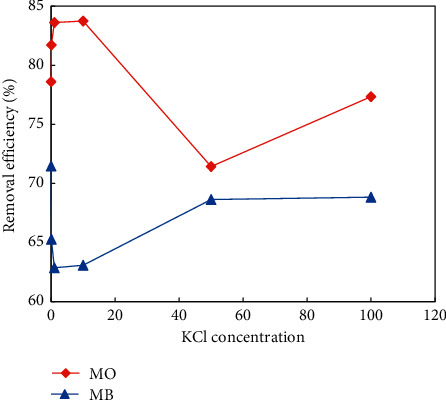
Influence of KCl on the adsorption of MB and MO by UiO-66-NO_2_.

To more precisely evaluate the impact of the electrostatic interaction, the adsorption of MB and MO was measured under the addition of different amounts of KCl. The presence of KCl in the solution increases the ionic strength. As can be seen, the addition of KCl has no great impact on the adsorption of both MB and MO, thus confirming the minor impact of the electrostatic interaction of these dyes with UiO-66-NO_2_. It is notable to mention that MO and MB were in the negative and positive form at the experiment condition known as the neutral condition because this pH was higher than pK_a_ of both MO (pK_a_ = 3.7) and MB (pK_a_ = 3.8). Moreover, the isoelectric point of UiO-66-NO_2_ was 4.6; thus, the surface of the material was negatively charged at a neutral condition. Therefore, as can be seen in Figure 9, at low KCl concentration, K^+^ ion neutralized the negative charge of the material surface that facilitated the adsorption of MO on the surface but enhanced the repulsion between MB and the surface of UiO-66-NO_2_. Consequently, the MO adsorption ability of the material enhanced, while the MB adsorption ability was in an opposite behavior.

At higher KCl concentration, the MO adsorption was remarkably decreased plausibly because of the interaction between the K^+^ ion and the negatively charged MO adsorbate, which reduced the affinity of MO and the surface. In the case of MB adsorption, the increase of KCl concentration limited the dissociation of MB molecules, which in turn reduced its repulsive interaction with the surface. As a result, the MO adsorption ability was reduced, while that of MB was increased.

## 4. Conclusion

The adsorption of methylene blue and methyl orange by UiO-66-NO_2_ nanoparticles was investigated. It was found that the adsorption of these dyes by UiO-66-NO_2_ takes place in both intraparticle and interparticle spaces. The kinetics of the adsorption fits moderately with the pseudo-first-order, but perfectly fits with pseudo-second-order models, and has a very good fit with the Elovich and intraparticle models. There is a slight difference in the adsorption mechanism of MB and MO onto UiO-66-NO_2_. However, for both dyes, the interaction between these solutes and the adsorbents is chemisorption, and the intraparticle diffusion is the rate-determining step. The adsorption capacity of methyl orange was greater than that of methylene blue. The reason might be due to the negative mesomeric effect of the -NO_2_ groups involved in the structure of UiO-66-NO_2_, which probably improves both *π*-*π* stacking and hydrogen bonds with the dye molecules. The hydrogen bonds have probably the predominant impact on the attraction between UiO-66-NO_2_ and the dye molecules.

## Figures and Tables

**Table 1:** Adsorption capacities of zirconium-based MOFs for MB and MO.

Absorbents	Dyes	*Q*_max_ (mg/g)	Reference
UiO-66	MO (mg/g)	39.42	[28]
UiO-66-NH_2_		28.97	[28]
UiO-66-NO_2_		142.9	This work

UiO-66	MB (mg/g)	90.88	[28]
UiO-66-NH_2_		96.45	[28]
UiO-66-NO_2_		41.7	This work

## Data Availability

The data used to support the findings of this study are included within the article.

## Supplementary Materials

Supplementary Materials Figure S1: TEM image of UiO-66-NO_2_. Figure S2: pseudo-first-order kinetic model of MB adsorption onto UiO-66-NO_2_. Figure S3: pseudo-first-order kinetic model of MO adsorption onto UiO-66-NO_2_. Figure S4: pseudo-second-order kinetic model of MB adsorption onto UiO-66-NO_2._ Figure S5: pseudo-second-order kinetic model of MO adsorption onto UiO-66-NO_2_. Figure S6: Elovich adsorption model of MB adsorption onto UiO-66-NO_2_ nanoparticles. Figure S7: Elovich adsorption model of MO adsorption onto UiO-66-NO_2_ nanoparticles. Figure S8: intraparticle adsorption model of MB adsorption onto UiO-66-NO_2_. Figure S9: intraparticle adsorption model of MO adsorption onto UiO-66-NO_2_.
