# Supplementary material for: Investigation into the Adsorption of Methylene Blue and Methyl Orange by UiO-66-NO2 Nanoparticles
Source: J Anal Methods Chem. 2021 Jun 12;2021:5512174. doi: 10.1155/2021/5512174 (PMC8216806; doi:10.1155/2021/5512174)
Supplement: Supplementary Materials — Figure S1: TEM image of UiO-66-NO2. Figure S2: pseudo-first-order kinetic model of MB adsorption onto UiO-66-NO2. Figure S3: pseudo-first-order kinetic model of MO adsorption onto UiO-66-NO2. Figure S4: pseudo-second-order kinetic model of MB adsorption onto UiO-66-NO2. Figure S5: pseudo-second-order kinetic model of MO adsorption onto UiO-66-NO2. Figure S6: Elovich adsorption model of MB adsorption onto UiO-66-NO2 nanoparticles. Figure S7: Elovich adsorption model of MO adsorption onto UiO-66-NO2 nanoparticles. Figure S8: intraparticle adsorption model of MB adsorption onto UiO-66-NO2. Figure S9: intraparticle adsorption model of MO adsorption onto UiO-66-NO2. [file 5512174.f1.zip › 5512174.f1/supporting information.docx]

**Supporting information**

# Investigation into the adsorption of methylene blue and methyl orange by UiO-66-NO_2_ nanoparticles

Hien Thi Dinh, Nam Trung Tran, Dai Xuan Trinh*

Faculty of Chemistry, VNU University of Science, Vietnam National University, Hanoi, Vietnam

*19 Le Thanh Tong str., Hoan Kiem Distr., Hanoi, Vietnam*

# Corresponding author:

# Dr. Dai Xuan Trinh

Email: [daitx@vnu.edu.vn](mailto:daitx@vnu.edu.vn)

Telephone: +84 978 999 977

**1. TEM images of UiO-66-NO_2_ nanoparticles**

Fig. S. 1 shows TEM images of the UiO-66-NO_2_ nanoparticles in which the particle size was homogeneous in the range of 45 – 65 nm.

| 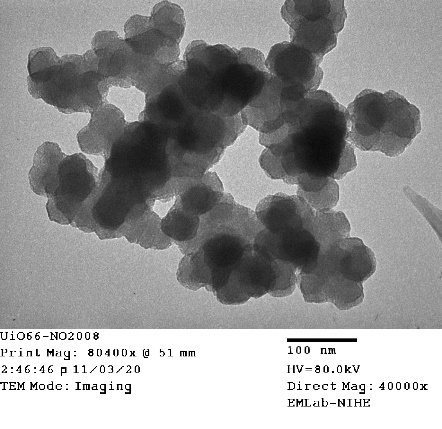 | 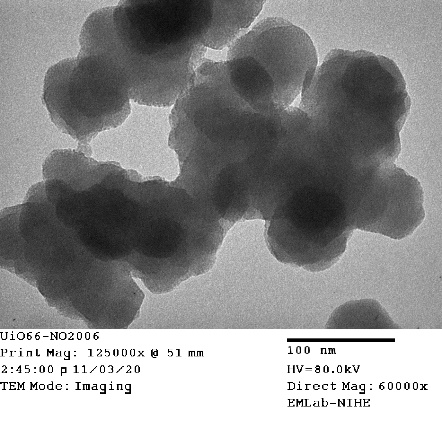 |
| --- | --- |

**Figure S. 1.** TEM image of UiO-66-NO_2_

**2.** **Pseudo first order kinetic model of MB and MO adsorption on UiO-66-NO_2_**

The kinetic models of the dye adsorption were conducted in three concentrations


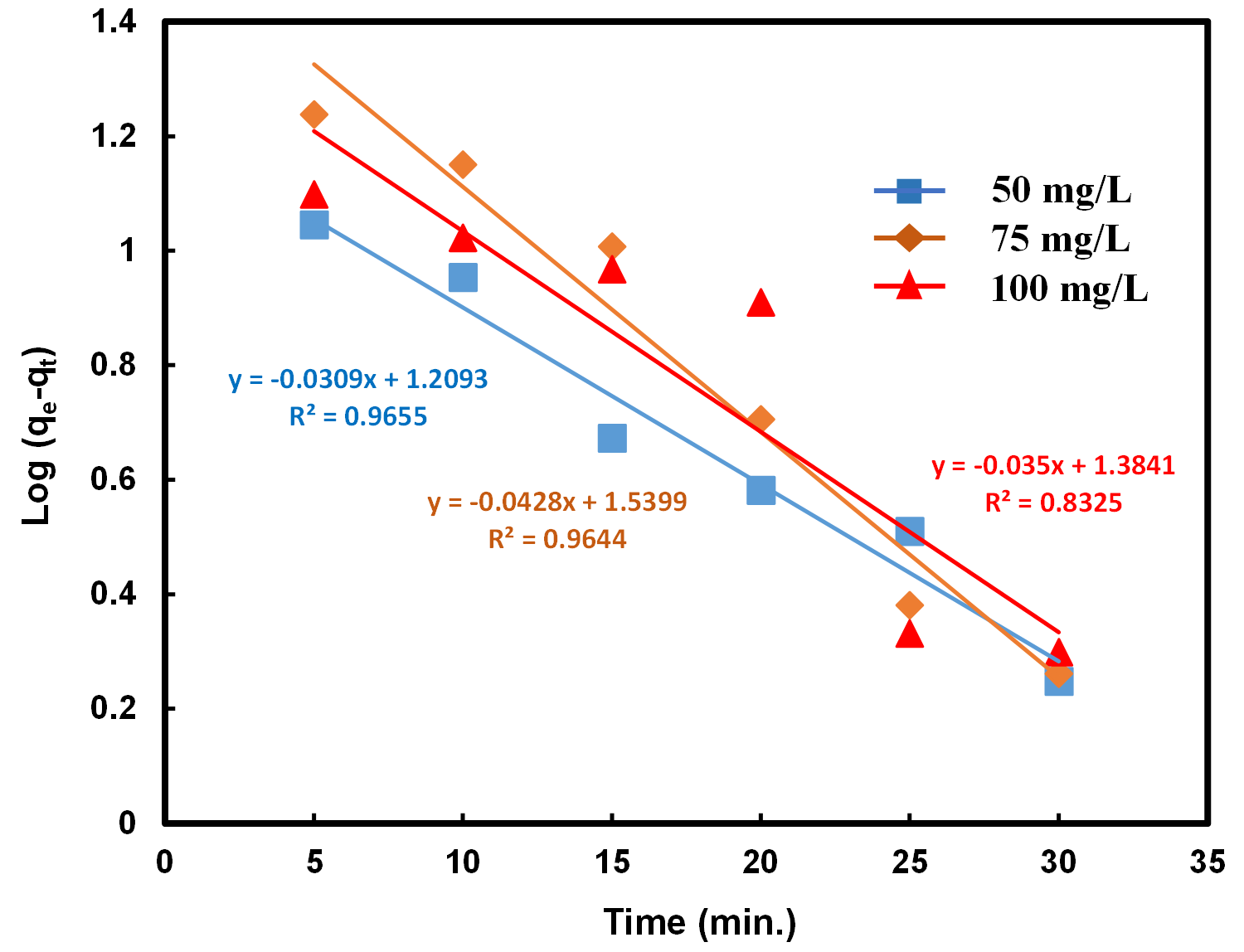


**Figure S. 2.** Pseudo first order kinetic model of MB adsorption on UiO-66-NO_2_


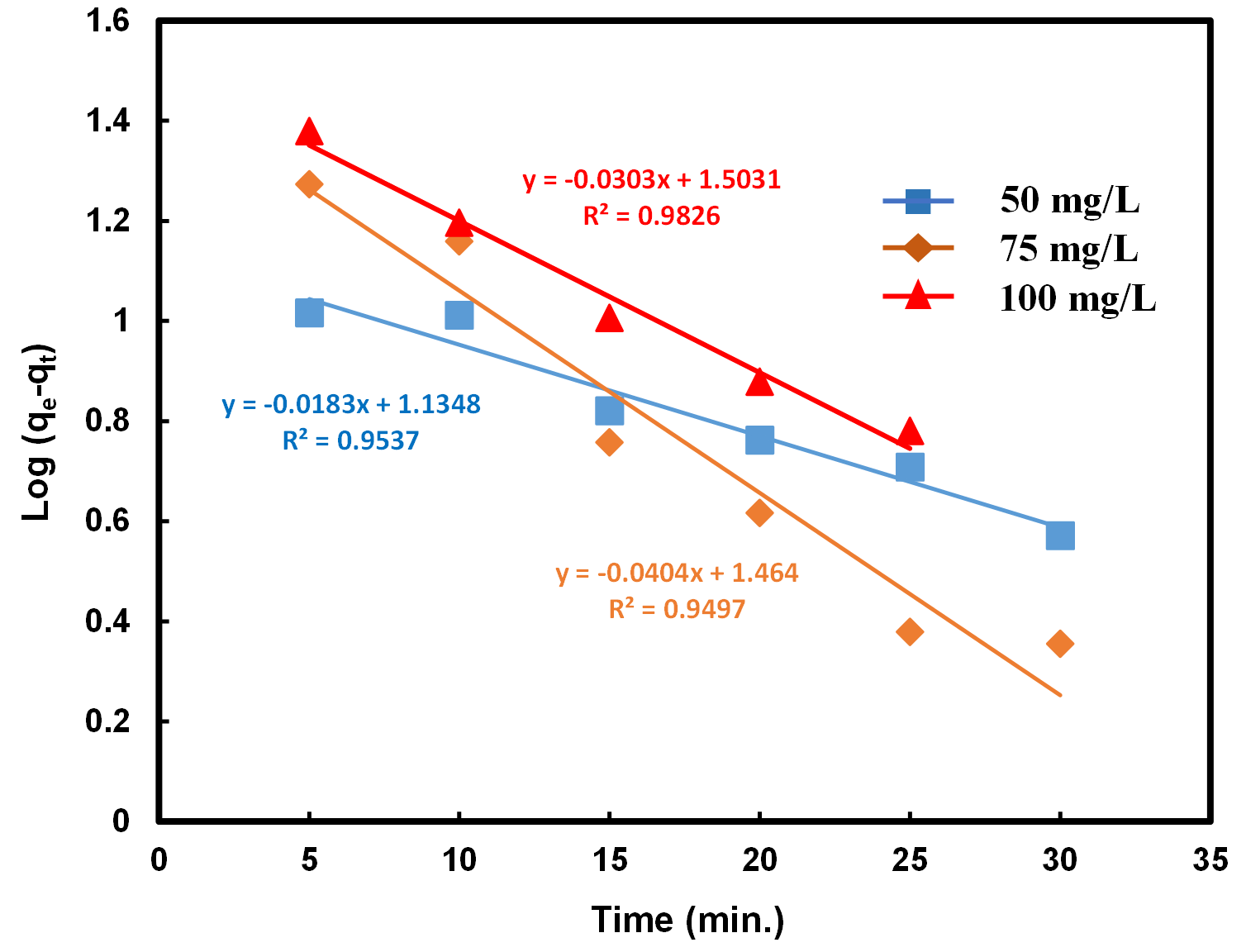


**Figure S. 3.** Pseudo first order kinetic model of MO adsorption on UiO-66-NO_2_

**
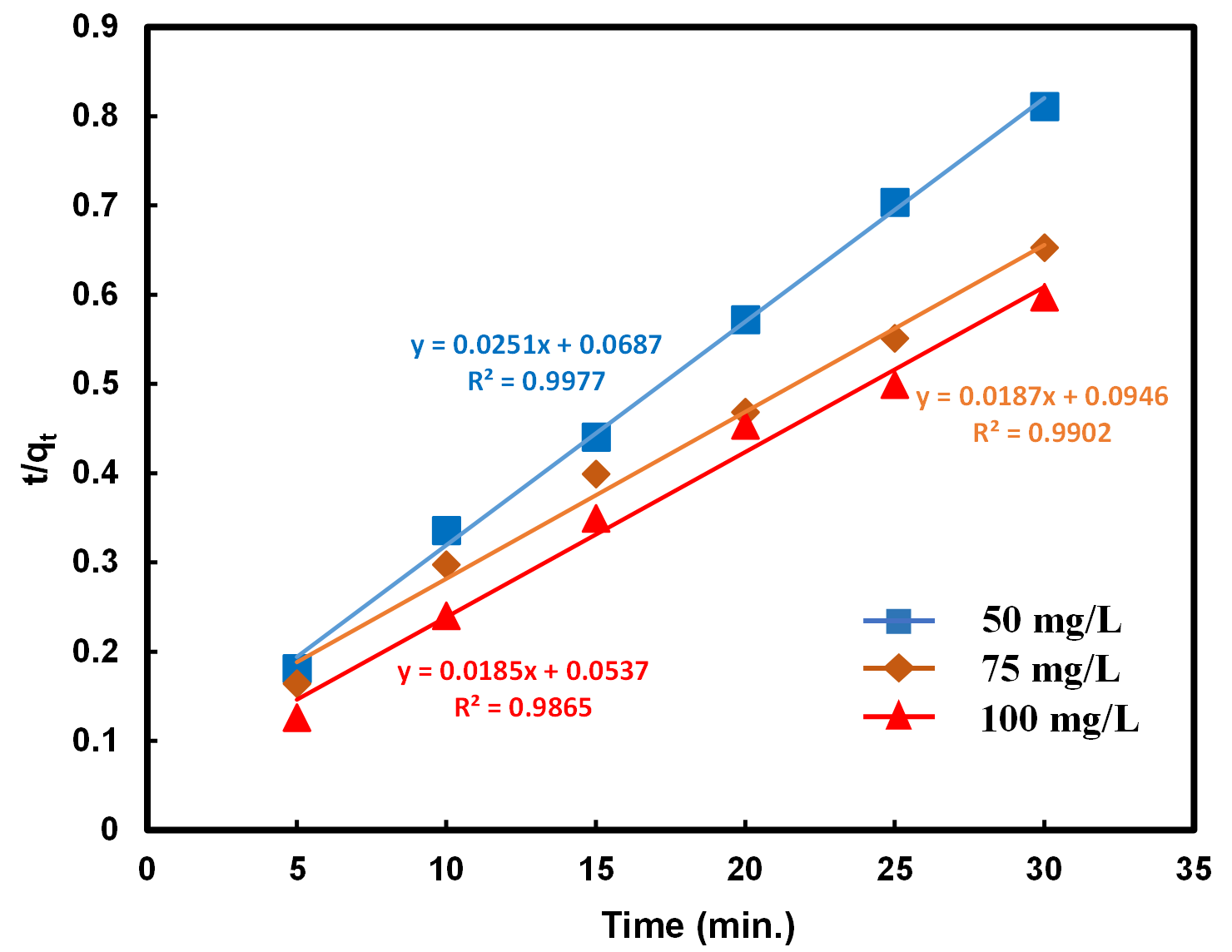
**

**Figure S. 4.** Pseudo second order kinetic model of MB adsorption on UiO-66-NO_2_

**
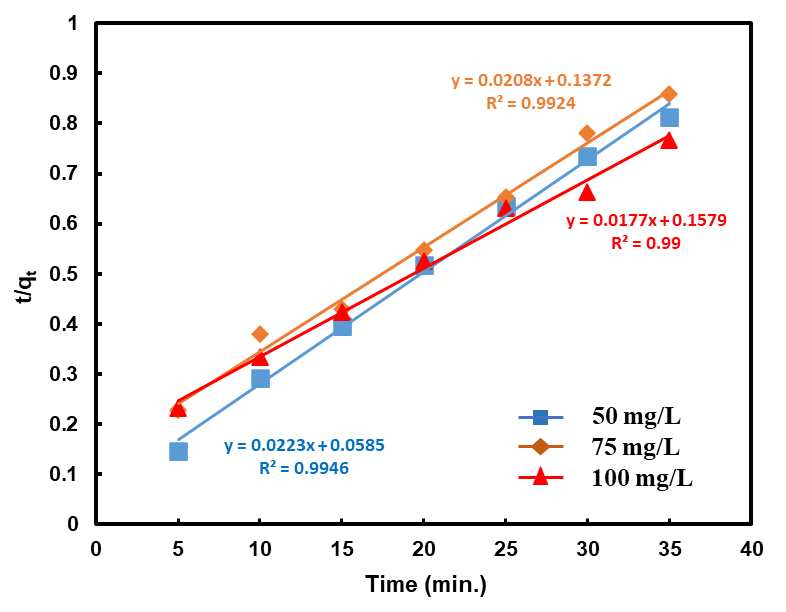
**

**Figure S. 5.** Pseudo second order kinetic model of MO adsorption on UiO-66-NO_2_


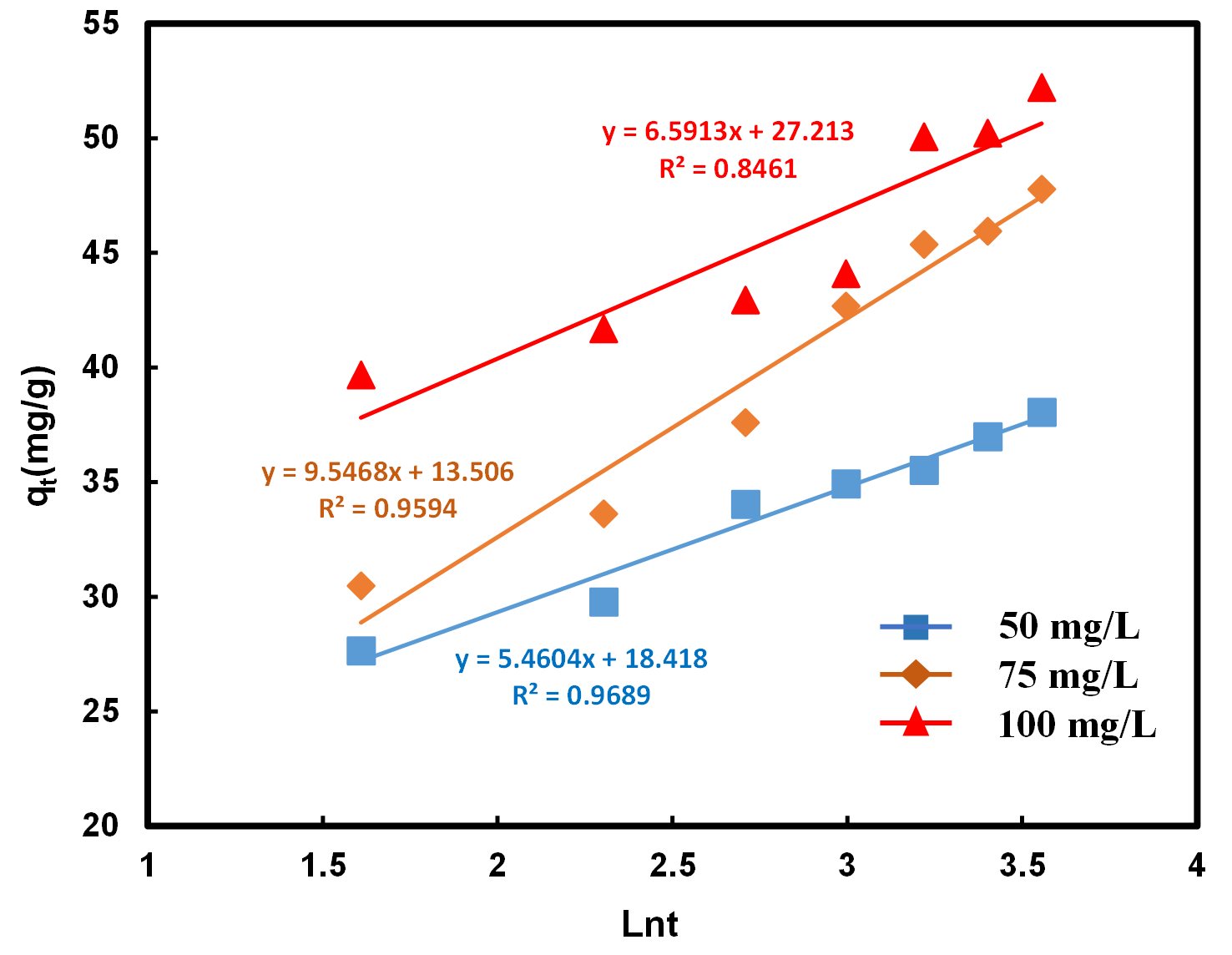


**Figure S. 6.** Elovich adsorption model of MB adsorption on UiO-66-NO­_2_ anoparticles

**
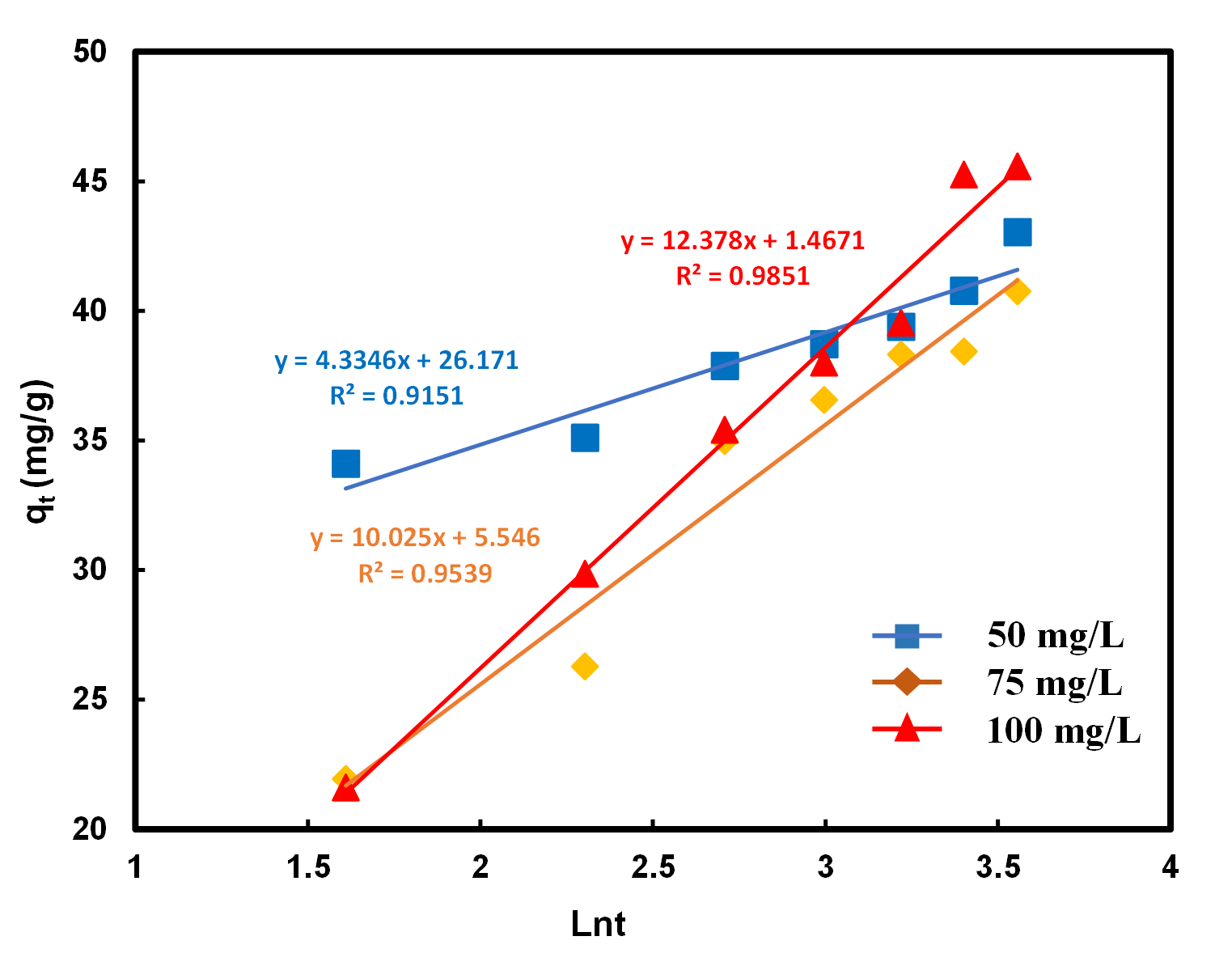
**

**Figure S. 7.** Elovich adsorption model of MO adsorption on UiO-66-NO­_2_ anoparticles


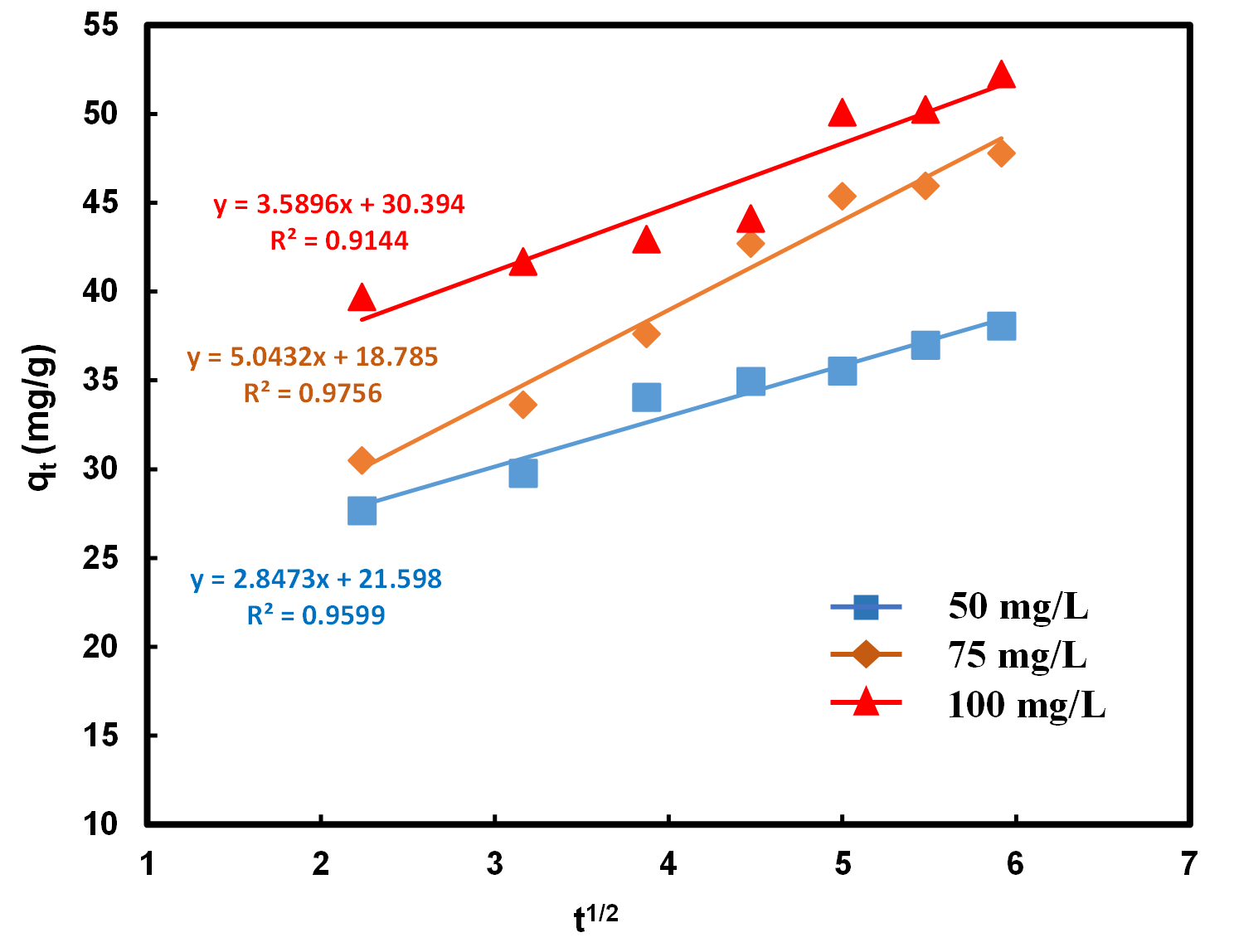


**Figure S. 8.** Intraparticle adsorption model of MB adsorption on UiO-66-NO_2_


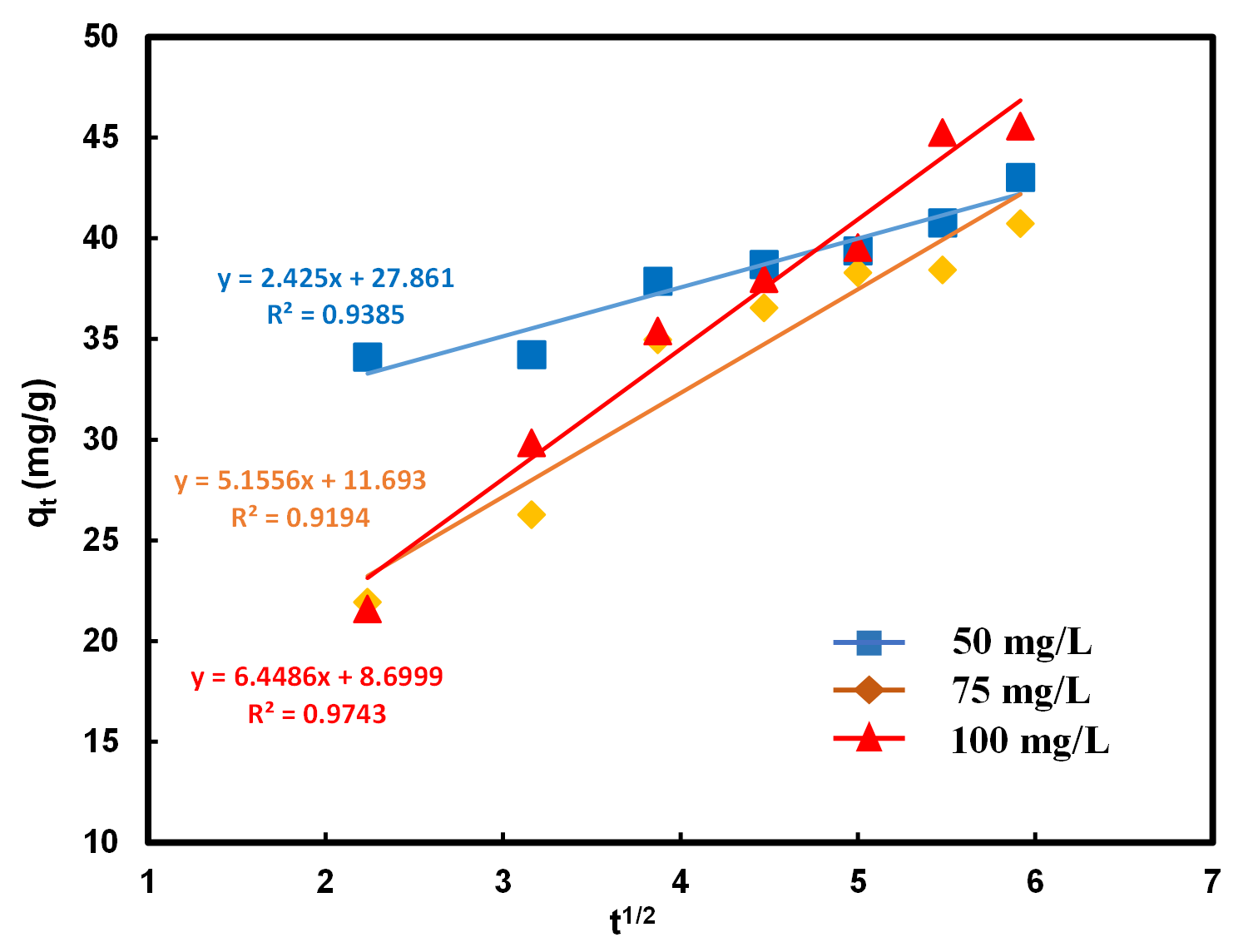


**Figure S. 9.** Intraparticle adsorption model of MO adsorption on UiO-66-NO_2_
