# Supplementary material for: Investigation into the Adsorption of Methylene Blue and Methyl Orange by UiO-66-NO2 Nanoparticles
Source: J Anal Methods Chem. 2021 Jun 12;2021:5512174. doi: 10.1155/2021/5512174 (PMC8216806; doi:10.1155/2021/5512174)
Supplement: Supplementary Materials — Figure S1: TEM image of UiO-66-NO2. Figure S2: pseudo-first-order kinetic model of MB adsorption onto UiO-66-NO2. Figure S3: pseudo-first-order kinetic model of MO adsorption onto UiO-66-NO2. Figure S4: pseudo-second-order kinetic model of MB adsorption onto UiO-66-NO2. Figure S5: pseudo-second-order kinetic model of MO adsorption onto UiO-66-NO2. Figure S6: Elovich adsorption model of MB adsorption onto UiO-66-NO2 nanoparticles. Figure S7: Elovich adsorption model of MO adsorption onto UiO-66-NO2 nanoparticles. Figure S8: intraparticle adsorption model of MB adsorption onto UiO-66-NO2. Figure S9: intraparticle adsorption model of MO adsorption onto UiO-66-NO2. [file 5512174.f1.zip › 5512174.f1/supporting information.pdf]

Supporting information

Investigation into the adsorption of methylene blue and methyl orange by UiO-66-NO_2 nanoparticles

Hien Thi Dinh, Nam Trung Tran, Dai Xuan Trinh*

Faculty of Chemistry, VNU University of Science, Vietnam National University, Hanoi, Vietnam

19 Le Thanh Tong str., Hoan Kiem Distr., Hanoi, Vietnam

Corresponding author:

Dr. Dai Xuan Trinh

Email: daitx@vnu.edu.vn

Telephone: +84 978 999 977

TEM images of UiO-66-NO_2 nanoparticles

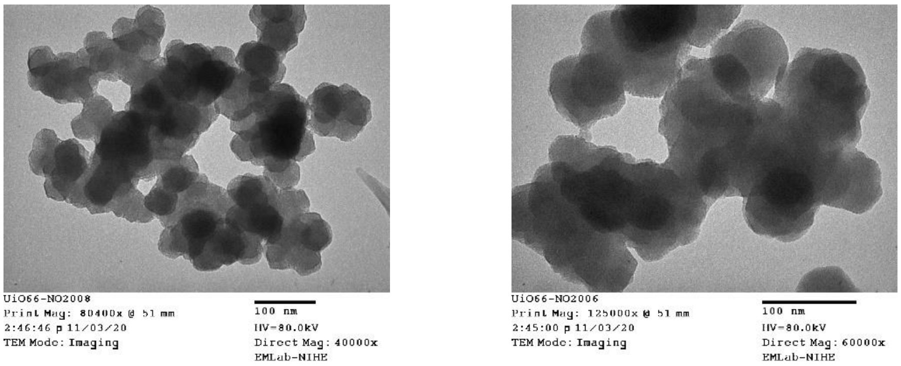

Figure S. 1. TEM image of UiO-66-NO_2

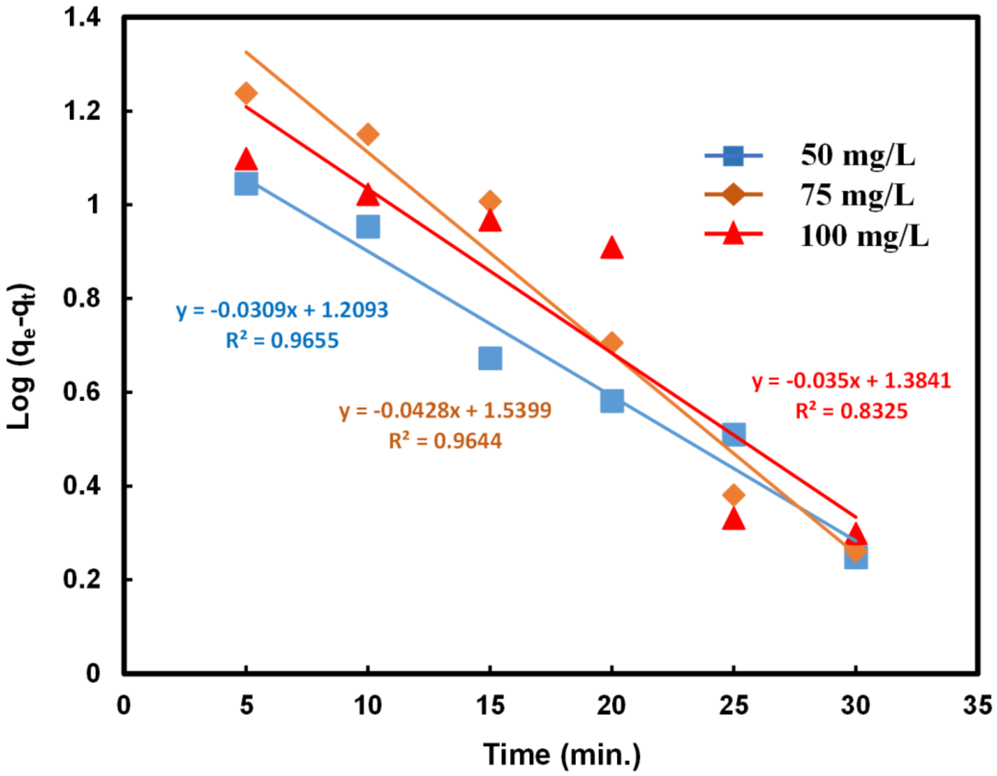

Figure S. 2. Pseudo first order kinetic model of MB adsorption on UiO-66-NO_2

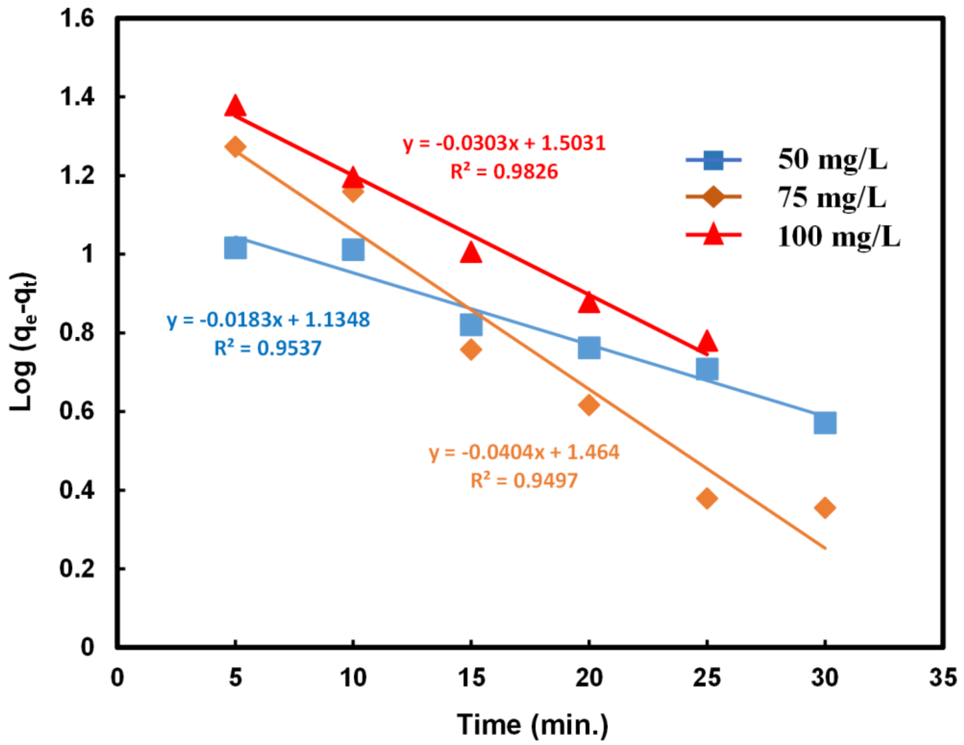

Figure S. 3. Pseudo first order kinetic model of MO adsorption on UiO-66-NO_2

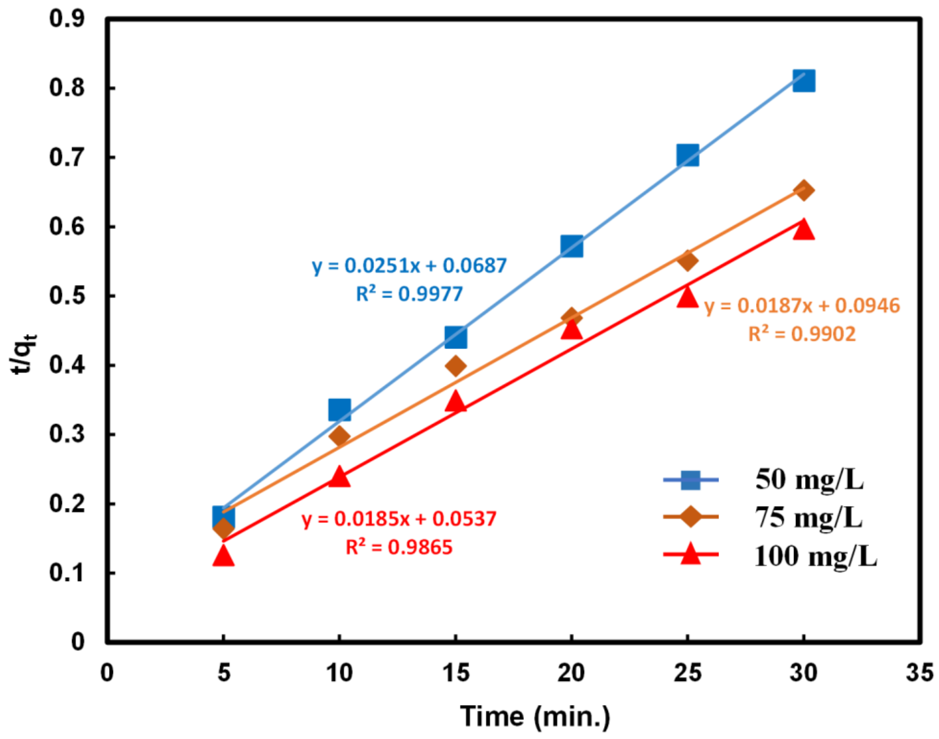

Figure S. 4. Pseudo second order kinetic model of MB adsorption on UiO-66-NO_2

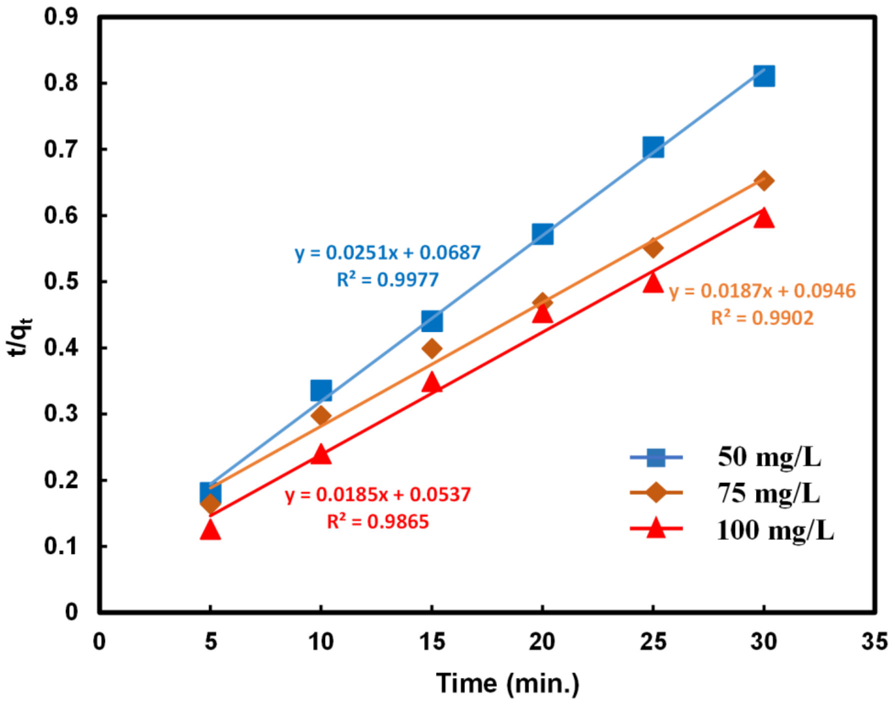

Figure S. 5. Pseudo second order kinetic model of MO adsorption on UiO-66-NO_2

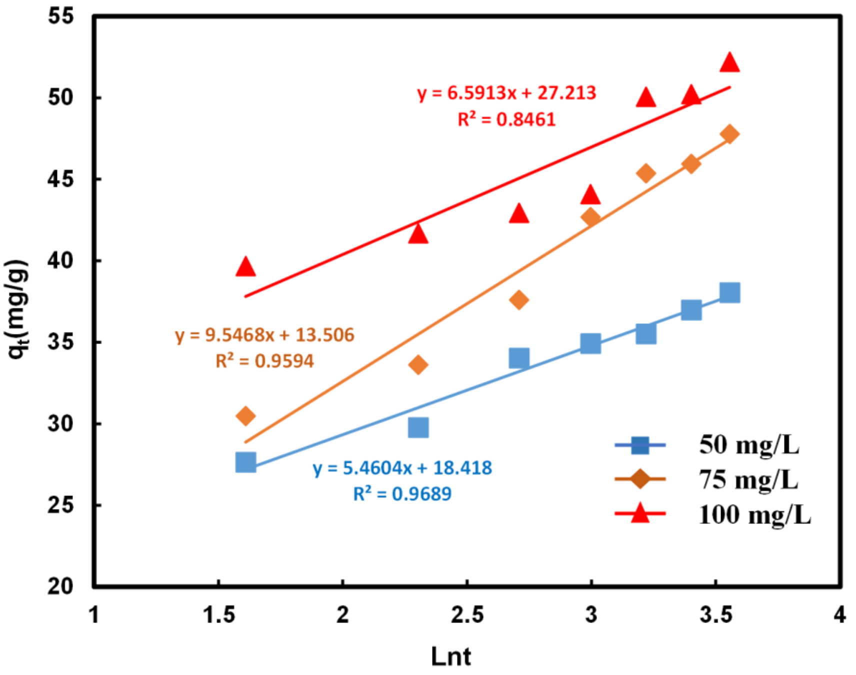

Figure S. 6. Elovich adsorption model of MB adsorption on UiO-66-NO_2 anoparticles

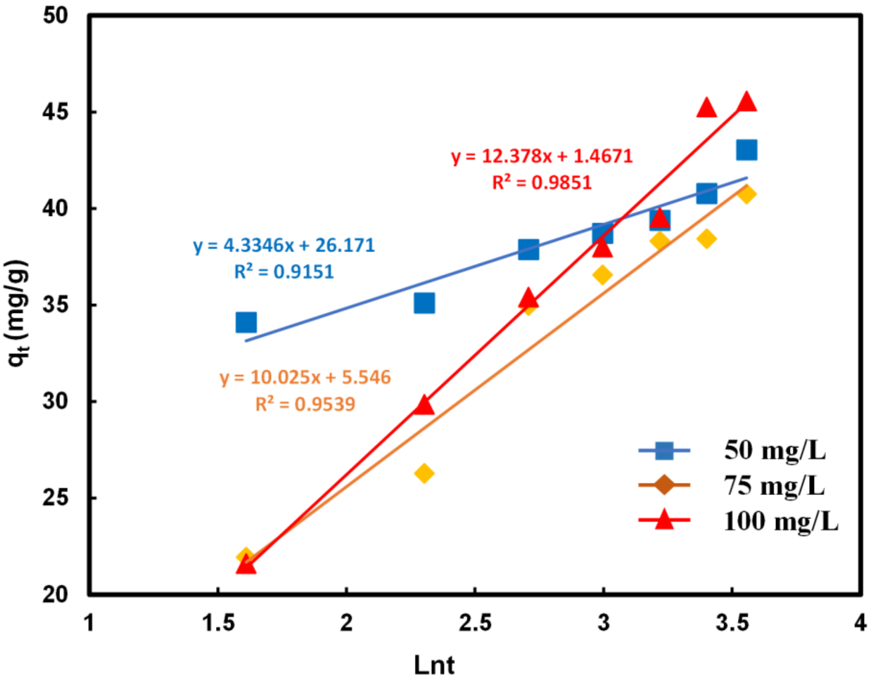

Figure S. 7. Elovich adsorption model of MO adsorption on UiO-66-NO_2 anoparticles

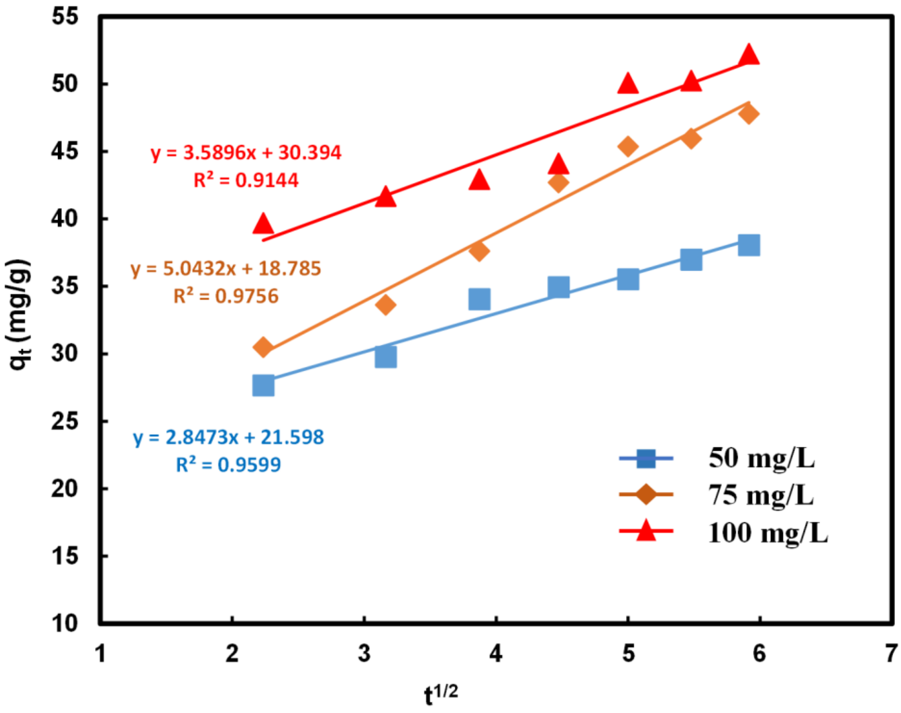

Figure S. 8. Intraparticle adsorption model of MB adsorption on UiO-66-NO_2

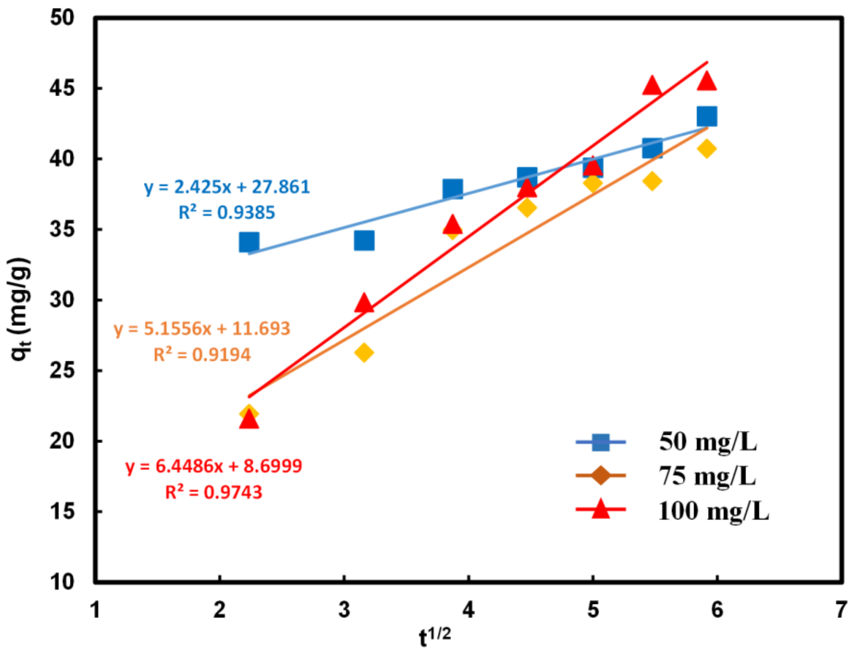

Figure S. 9. Intraparticle adsorption model of MO adsorption on UiO-66-NO_2
